# Mechanical Acupuncture at ST36 Attenuates Inflammatory Pain Involving TRPV1 Signaling in Mice

**DOI:** 10.3390/ijms26178534

**Published:** 2025-09-02

**Authors:** Suk-Yun Kang, Se Kyun Bang, Su Yeon Seo, Seong Jin Cho, Kwang-Ho Choi, Sangeun Han, Yeonhee Ryu

**Affiliations:** Korea Institute of Oriental Medicine, Daejeon 34054, Republic of Korea; sy8974@kiom.re.kr (S.-Y.K.); sichosi@kiom.re.kr (S.K.B.); ssy1025@kiom.re.kr (S.Y.S.); ipcng@kiom.re.kr (S.J.C.); ddoongho@kiom.re.kr (K.-H.C.); sadgc0303@kiom.re.kr (S.H.)

**Keywords:** mechanical acupuncture instrument, alternative therapy, inflammatory pain, acute pain, analgesic, TRPV1, glial cells

## Abstract

We recently developed a mechanical acupuncture instrument (MAI) that applies mechanical stimulation to acupuncture points in effectively treating hypertension and addiction in animal models. However, its analgesic effect on inflammatory pain remains unclear. Here, we aimed to determine the optimal duration of MAI treatment at any given acupuncture point to improve analgesic effects. Adult male ICR mice (20–25 g, 6 weeks old, n = 6 per group) were used to evaluate whether MAI administration or TRPV1 (transient receptor potential vanilloid 1) inhibition had analgesic effects. Then, we investigated whether it affected TRPV1 expression and glial cells in the spinal cord of mice. The capsaicin test was used to identify the most effective acupoints and optimal treatment times for MAI. Additionally, we induced inflammatory pain in mice by administering a 2% carrageenan via intraplantar injection. To assess the analgesic effects of MAI treatment and TRPV1 inhibition, we evaluated pain-related behavior using von Frey filaments and a thermal stimulator applied to the hind paw. MAI treatment significantly suppressed pain-related behaviors. In particular, paw-licking duration was markedly reduced in the group treated with MAI for 60 s at ST36 compared to the capsaicin-treated group (*p* < 0.05), suggesting a robust analgesic effect. Additionally, MAI and capsazepine administration significantly attenuated carrageenan-induced mechanical allodynia and thermal hyperalgesia compared to the carrageenan-only group (*p* < 0.05 to *p* < 0.001). Additionally, MAI treatment and capsazepine administration effectively suppressed the carrageenan-induced upregulation of TRPV1 and glial cells in the spinal cord. In conclusion, our findings show that MAI administration at ST36 significantly alleviated inflammatory pain and was associated with downregulation of TRPV1 expression and microglial activation in the spinal cord. The present findings suggest that TRPV1 signaling is involved in the analgesic effects of mechanical acupuncture; however, a direct causal relationship has yet to be established.

## 1. Introduction

Inflammatory pain is a distinct and multifaceted subtype of nociceptive pain that arises following tissue injury or infection, resulting from the interplay between immune activation, peripheral sensitization, and central nervous system (CNS) modulation. When tissues are damaged or exposed to pathogens, a cascade of pro-inflammatory mediators—including cytokines such as interleukin-6 (IL-6) and tumor necrosis factor-α (TNF-α), prostaglandins, lipid mediators, and neuropeptides—are released, leading to the recruitment and activation of immune cells at the injury site [1]. These substances sensitize peripheral nociceptors by lowering their activation threshold and contribute to enhanced excitability of pain pathways within the CNS, thereby complicating the clinical management of inflammatory pain [2]. While acute inflammatory pain plays a protective role by promoting withdrawal from harmful stimuli and supporting tissue repair, this response can become maladaptive when dysregulated, leading to persistent pain and the development of chronic inflammatory conditions. Such states are characterized by sustained neuroimmune activation, ongoing peripheral and central sensitization, motor dysfunction, and emotional disturbances which collectively result in a significant reduction in quality of life. Chronic inflammatory pain contributes substantially to the global burden of diseases such as rheumatoid arthritis, inflammatory bowel disease, and neuropathic pain syndromes, and remains a major challenge in clinical and public health settings [3,4,5].

In experimental inflammatory pain models, such as those induced by carrageenan or capsaicin, the transient receptor potential vanilloid 1 (TRPV1) function and expression are markedly upregulated in both peripheral terminals and central projections of nociceptors. Wang et al. reported that increased TRPV1 activity facilitates the release of neuropeptides—including substance P and calcitonin gene-related peptide (CGRP)—which drive further neuroimmune activation, microglial and astrocyte response, and central sensitization [6,7,8]. Recent studies have demonstrated that TRPV1 is upregulated under inflammatory conditions, and that it interacts with other key pain mediators, such as the N-methyl-D-aspartate (NMDA) receptor. The inhibition of spinal TRPV1 reduces NMDA receptor 2 B phosphorylation and glutamate release in the spinal cord, thereby diminishing central sensitization and attenuating pain behavior in animal models of inflammatory pain [9]. Other studies have shown that TRPV1 function is potentiated by inflammatory mediators, which can lower its activation threshold and increase its expression at both the transcriptional and translational levels [6]. This makes TRPV1 a sensor as well as an amplifier of inflammatory pain and an attractive target for analgesic strategies [7,10].

Acupuncture is increasingly recognized as an effective analgesic modality in both human and veterinary medicine, particularly for chronic and inflammation-related pain, although its underlying mechanisms remain incompletely understood and are still largely explained within the framework of traditional medical philosophies. Recent studies have begun to bridge this gap by demonstrating that acupuncture influences neuroimmune mechanisms, such as glial cell modulation and ion channel regulation. One of the key transducers thought to mediate acupuncture-induced analgesia is the TRPV1 channel, well-established for its role in nociceptive signaling and peripheral sensitization. Acupuncture, particularly at the Zusanli (ST36) acupoint, has been shown to alleviate both acute and chronic pain through multiple mechanisms, including the modulation of peripheral nociceptor sensitivity, inhibition of pro-inflammatory cytokine release, and attenuation of central sensitization via the suppression of spinal glial activation (microglia and astrocytes) [9,11]. The development of advanced acupuncture techniques, such as nanoporous needles and mechanical acupuncture instrument (MAI), has further enhanced therapeutic effects by increasing local tissue deformation and engaging mechanosensitive ion channels more effectively [12]. Mechanical stimulation at acupoints is transduced into neuroelectrical and biochemical signals via ion channels that contribute to mechano-nociceptive processing under pathological conditions, including members of the TRP family such as TRPV1 and acid-sensing ion channels [13]. Recent advances, such as MAI, enable controlled repetitive stimulation, presenting an opportunity to explore this relationship in a reproducible model.

This study is based on the hypothesis that mechanical acupuncture at the ST36 acupoint exerts analgesic effects in inflammatory pain by modulating TRPV1 signaling and suppressing spinal glial activation. Therefore, the primary objective of this study was to determine the analgesic efficacy of MAI treatment at different acupoints and durations in a well-established mouse model of carrageenan-induced inflammatory pain. Specifically, we aimed to investigate whether MAI-mediated stimulation at the ST36 point could alleviate inflammatory pain-related behaviors more effectively than stimulation at other points or non-acupoints, as quantified by spontaneous pain tests and evoked pain behaviors. To elucidate the mechanisms underlying this analgesic effect, we examined the involvement of spinal TRPV1 signaling and glial cell activation.

## 2. Results

### 2.1. Anti-Nociceptive Effect of Treating Different Acupuncture Points in Capsaicin-Induced Spontaneous Pain Test

**Figure 1:**
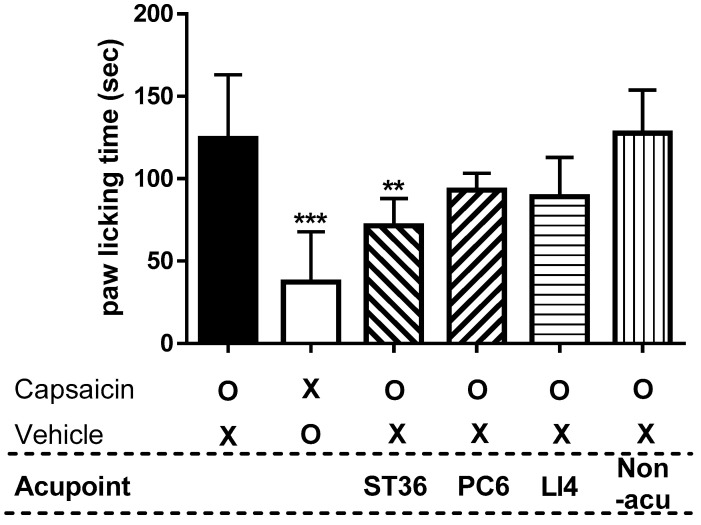
Paw-licking time after mechanical acupuncture instrument (MAI) treatment on various acupuncture points related to pain in the capsaicin-induced spontaneous pain mouse model. All experimental groups, except for the vehicle control, received intraplantar capsaicin injections (1.6 µg/paw) to induce pain-related behavior. The first two bars represent groups that did not receive any acupoint or non-acupoint stimulation: the capsaicin group received only capsaicin injection, while vehicle group received only saline injection, without MAI or acupuncture intervention. All remaining groups (ST36, PC6, LI4, Non-acu) received capsaicin injection followed by mechanical acupuncture stimulation at the designated site. Intraplantar injection of capsaicin (capsaicin, n = 6) elicited nociceptive paw-licking behavior, whereas animals receiving saline (vehicle, n = 6) exhibited significantly reduced paw-licking responses compared to the capsaicin group (*** *p* < 0.001)**.** In the experimental group treated at ST36 acupoint (ST36, n = 6), the capsaicin-induced paw-licking time was significantly suppressed compared to the capsaicin-treated group (** *p* < 0.01). The PC6, LI4, and non-acupoint groups (PC6, LI4, and Non-acu, n = 6, respectively) exhibited no significant change in paw-licking time. Symbols: “O” represents treated; “X” represents untreated.

A capsaicin test was performed to determine which of the various acupuncture points related to pain behavior had the best anti-nociceptive efficacy. As shown in Figure 1, capsaicin, a TRPV1 channel activator, was used to induce paw-licking behavior, which is characteristic of nociception, and the time required to lick the paw was measured. Intraplantar capsaicin injection resulted in a substantial increase in paw-licking behavior (124.3 ± 38.8 s), significantly exceeding the response observed in the vehicle-treated group (37.3 ± 30.4 s, *p* < 0.001).

The experimental group where MAI was administered to the ST36 acupuncture point exhibited a decrease in paw-licking time to 71.3 ± 16.7 s compared to that of mice injected with capsaicin into the paw (*p* < 0.01). When MAI was administered to the PC6 and LI4 acupuncture points, the capsaicin-induced paw-licking times were 93.0 ± 10.2 s and 89.2 ± 23.7 s, respectively, with no statistically significant difference compared to the capsaicin-injected group. Additionally, the experimental group where MAI was applied to the non-acupuncture site did not exhibit reduced capsaicin-induced paw-licking time (127.7 ± 26.0 s).

### 2.2. Time-Dependency of MAI in Capsaicin-Induced Spontaneous Pain Test

**Figure 2:**
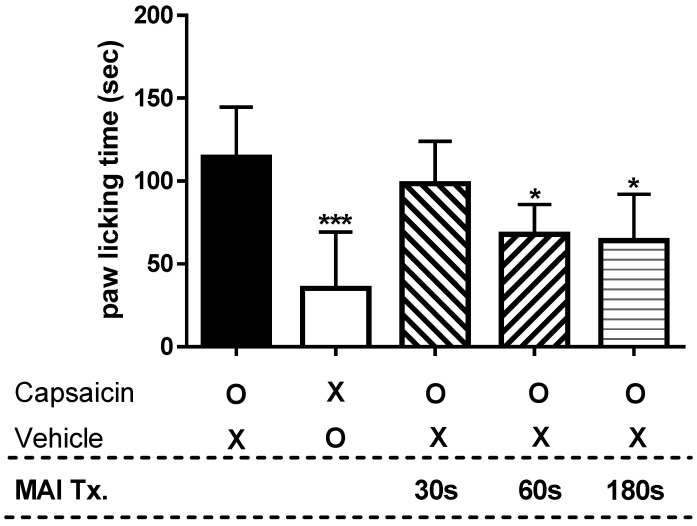
Paw-licking time according to the MAI treatment time in the capsaicin-induced foot pain mouse model. All experimental groups, except for the vehicle control, received intraplantar capsaicin injections (1.6 µg/paw) to induce pain-related behavior. The “capsaicin” group received the capsaicin injection without subsequent MAI treatment, while the other groups were treated with MAI at the ST36 acupuncture point for the specified durations immediately after capsaicin injection. Intraplantar injection of capsaicin (Capsaicin, n = 6) induced nociceptive characteristic paw-licking time, which decreased significantly in saline-injected animals (Vehicle, n = 6) (*** *p* < 0.001). The experimental group treated with MAI for 30 s (MAI 30 s, n = 6) showed no significant change in capsaicin-induced paw-licking time compared to the capsaicin-treated group. However, in the experimental group treated for 60 and 180 s (MAI 60 s and 180 s, n = 6, respectively), the capsaicin-induced paw-licking time was significantly suppressed compared to the experimental group treated with capsaicin (* *p* < 0.05). Symbols: “O” represents treated; “X” represents untreated.

A capsaicin test was performed to determine the anti-nociceptive effect of MAI, a new acupuncture point stimulator, according to treatment time. Figure 2 shows the paw-licking time measured to determine how long it takes to treat pain induced by capsaicin with MAI to achieve an anti-nociceptive effect. Intraplantar injection of capsaicin elicited a paw-licking response lasting 114.3 ± 30.2 s, which was significantly greater than that observed in the vehicle-treated group (35.3 ± 33.9 s, *p* < 0.001). In the experimental group treated with MAI for 30 s, capsaicin-induced paw-licking duration was 98.3 ± 25.6 s, showing no statistically significant difference compared with the capsaicin-only group. On the other hand, the experimental groups treated with MAI for 60 and 180 s showed a significant decrease compared to the experimental group treated with capsaicin at 67.8 ± 18.2 s and 63.8 ± 27.7 s, respectively (paw-licking time, in seconds).

### 2.3. Anti-Nociceptive Effect of MAI Treatment in Carrageenan-Induced Pain Behavior

**Figure 3:**
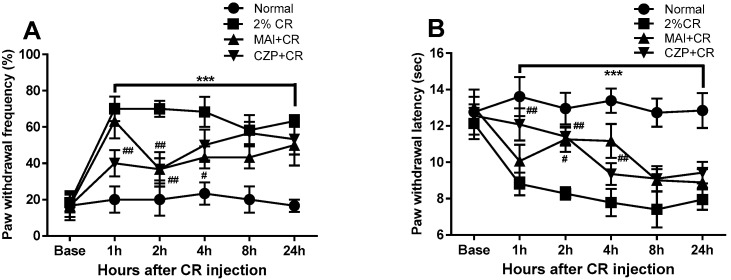
Anti-nociceptive effects of MAI treatment on mechanical allodynia (**A**) and thermal hyperalgesia (**B**) in carrageenan-treated mice. The intraplantar carrageenan injection group (2% CR, n = 6) exhibited significant alterations in paw withdrawal frequency and latency in the hind paws from 1 to 24 h post-injection, highlighting the robust induction of mechanical allodynia and thermal hyperalgesia by carrageenan (*** *p* < 0.001 vs. normal animals; Normal, n = 6). Mechanical allodynia induced by carrageenan was significantly attenuated at 2 and 4 h in the MAI-treated group (MAI + CR, n = 6) compared to mice with carrageenan-induced inflammatory pain (^#^ *p* < 0.05 and ^##^ *p* < 0.01). For thermal hyperalgesia, paw withdrawal latency was significantly increased at 2 and 4 h following carrageenan injection in the MAI-treated group compared to the carrageenan-only group (^#^ *p* < 0.05 and ^##^ *p* < 0.01). The capsazepine administration group (CZP + CR, n = 6) showed significantly suppressed carrageenan-induced mechanical allodynia and thermal hyperalgesia at 1 and 2 h compared with those in carrageenan-treated mice (^##^ *p* < 0.01).

Carrageenan administration via intraplantar injection resulted in significant mechanical allodynia and thermal hyperalgesia, as illustrated in Figure 3. The carrageenan-injected group (2% CR) exhibited a significant increase in paw withdrawal frequency (%) in response to mechanical stimuli compared to the normal animals (normal) from 1 h to 24 h after carrageenan administration in the ipsilateral hind paws (*p* < 0.001, Figure 3A). Carrageenan-treated animals exhibited a significant reduction in paw withdrawal latency in response to thermal stimuli from 1 to 24 h post-injection, compared to normal animals (*p* < 0.001, Figure 3B).

MAI treatment produced a temporary anti-nociceptive effect on mechanical allodynia and thermal hyperalgesia following carrageenan injection. MAI-treated animals (MAI + CR) showed a significant reduction in the increase in paw withdrawal frequency (%) induced by carrageenan at 2 and 4 h compared to carrageenan-induced inflammatory pain animals (*p* < 0.05, *p* < 0.01, Figure 3A). Regarding paw withdrawal latency (s) to thermal stimuli, MAI-treated animals showed an anti-hyperalgesic effect at 2 and 4 h compared to animals with carrageenan-treated inflammatory pain (*p* < 0.05, *p* < 0.01, Figure 3B).

Through previous experiments, we determined that 1 μg of capsazepine was an appropriate concentration to produce analgesic effects [9]. Capsazepine-treated animals (CZP + CR) showed significantly reduced carrageenan-induced mechanical allodynia and thermal hyperalgesia 1 and 2 h after carrageenan injection compared to animals with carrageenan-treated inflammatory pain (*p* < 0.01).

### 2.4. Expression of TRPV1 and Phosphorylated NR2B (pNR2B) in the Spinal Dorsal Horn

**Figure 4:**
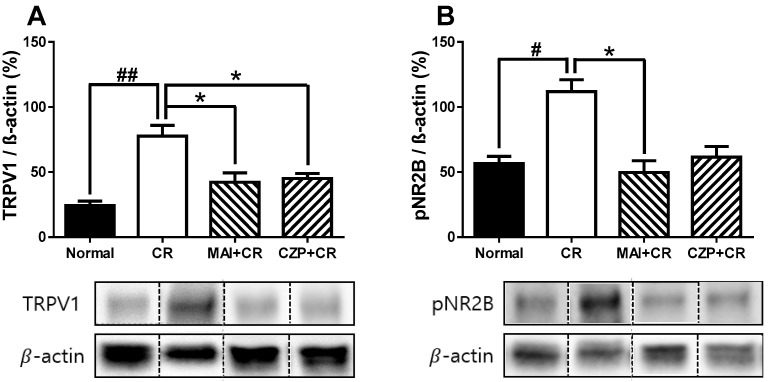
Effects of MAI treatment on transient receptor potential vanilloid 1 (TRPV1) (**A**) and pNR2B (**B**) expression in the lumbar spinal cord of carrageenan-treated mice. Carrageenan-injected animals (CR, n = 6) exhibited significantly elevated TRPV1 expression in the spinal cord compared to normal animals (Normal, n = 6; **^##^** *p* < 0.01). In contrast, TRPV1 expression was significantly reduced in both the MAI-treated group (MAI + CR, n = 6) and the intrathecal capsazepine-treated group (CZP + CR, n = 6), relative to the carrageenan-only group (* *p <* 0.05). Spinal pNR2B expression was significantly elevated following carrageenan injection compared to normal mice (**^#^** *p* < 0.05), whereas MAI-treated mice showed a marked reduction in pNR2B expression relative to the carrageenan-treated group (* *p* < 0.05).

Western blot analysis confirmed the effects of MAI and intrathecal capsazepine treatment on spinal TRPV1 and pNR2B expression at 2 h following carrageenan injection. Following injection, TRPV1 and pNR2B expression levels in the spinal dorsal horn were significantly elevated relative to those in normal controls (*p* < 0.01 and *p* < 0.05, respectively; Figure 4A,B). MAI treatment significantly suppressed carrageenan-induced upregulation of TRPV1 and pNR2B expression in the spinal dorsal horn (*p* < 0.05), indicating a strong anti-nociceptive effect of MAI in the context of carrageenan-induced inflammatory pain. Intrathecal pretreatment with capsazepine decreased spinal TRPV1 expression (*p* < 0.05) but had no effect on the spinal expression level of pNR2B.

### 2.5. Effect of MAI Treatment on Plasma TRPV1 Concentration

**Figure 5:**
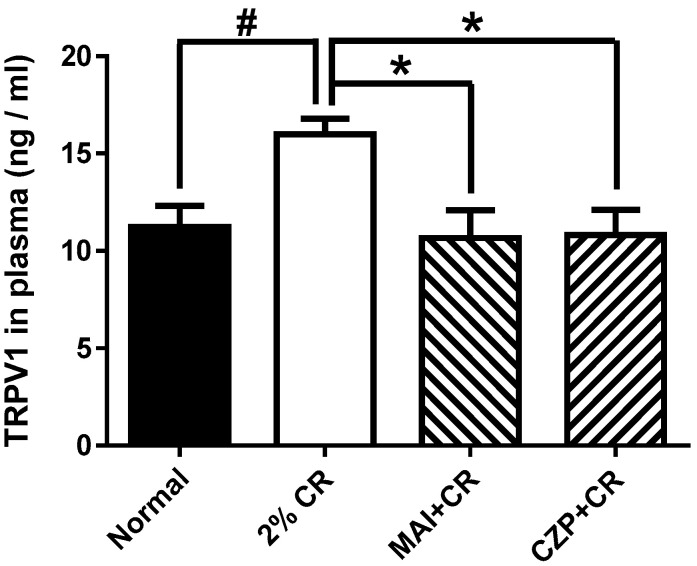
The effect of MAI treatment on plasma TRPV1 in mice with carrageenan-induced inflammatory pain. The mice with inflammatory pain (2% CR, n = 6) showed increased plasma TRPV1 levels compared with the normal animal group (Normal, n = 6, ^#^ *p* < 0.05). MAI (MAI + CR, n = 6) and capsazepine (CZP + CR, n = 6) administration significantly decreased the plasma TRPV1 level compared to animals with carrageenan-induced inflammatory pain (* *p* < 0.05). The results are shown as means ± SEM of the plasma TRPV1 levels in each group.

Next, we measured the level of TRPV1 in the plasma using ELISA kits (MyBioSource, San Diego, CA, USA). As shown in Figure 5, the plasma TRPV1 level in the normal group was 11.25 ± 2.62 ng/mL. In carrageenan-treated mice (2% CR), the peptide level increased to 15.99 ± 1.94 ng/mL, which was significantly higher than that observed in the normal group (*p* < 0.05). Plasma TRPV1 levels observed in mice treated with MAI at the ST36 acupuncture point (MAI + CR) decreased to 10.67 ± 3.49 ng/mL compared to those in mice injected with carrageenan (*p* < 0.05). On the other hand, the plasma TRPV1 level in the capsazepine intrathecal injection group (CZP + CR) was 10.82 ± 3.16 ng/mL, which was statistically decreased compared to the inflammatory pain group (*p* < 0.05).

### 2.6. Iba1 and GFAP Expression in the Spinal Dorsal Horn

**Figure 6:**
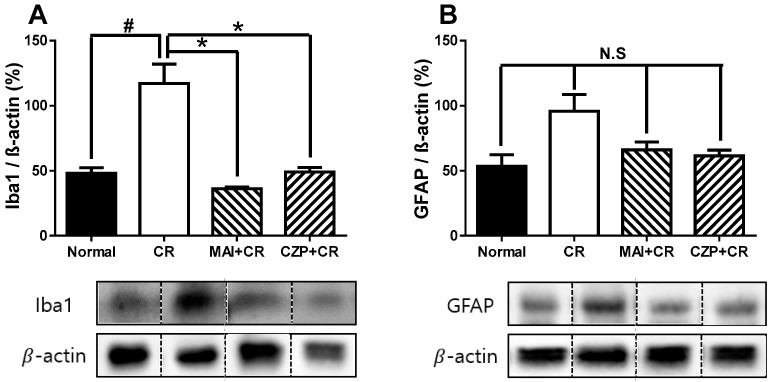
The effect of MAI treatment on microglia (**A**) and astrocytes (**B**) in the lumbar spinal cord of carrageenan-treated mice. Carrageenan-injected animals (CR, n = 6) showed significantly increased Iba1 (microglial marker) expression in the spinal cord compared to the normal group (Normal, n = 6, **^#^** *p* < 0.05), while MAI-treated animals (MAI + CR, n = 6) and intrathecal capsazepine-injected animals (CZP + CR, n = 6) exhibited significantly reduced Iba1 expression in the spinal cord compared to the carrageenan-injected group (* *p* < 0.05). GFAP expression in the spinal cord did differ significantly among the normal, carrageenan injection, MAI treatment, and capsazepine treatment groups. “N. S.” indicates “not significant” statistical differences (*p* > 0.05) according to the specified post hoc tests.

To determine how MAI treatment affected the abundance of glia, astrocytes, and microglia, Western blotting was performed on the dorsal horn of the lumbar spinal cord dorsal horn 2 h after carrageenan injection. Carrageenan injection significantly increased the number of microglia in the spinal dorsal horn compared to normal animals (*p* < 0.05) (Figure 6A), and MAI treatment at ST36 suppressed carrageenan-enhanced Iba1 (microglia marker) expression (*p* < 0.05), suggesting that MAI exerts a potent anti-nociceptive effect on carrageenan-induced inflammatory pain. Intrathecal capsazepine-injected animals also showed a significant decrease in Iba1 expression in the spinal cord dorsal horns compared to that of normal animals (*p* < 0.05). As shown in Figure 6B, the expression of GFAP (glial fibrillary acidic protein, an astrocyte marker) in the spinal cord did not decrease under the suppressive effect of MAI or intrathecal capsazepine treatment.

## 3. Discussion

Through this study, we demonstrated that mechanical stimulation of the ST36 acupoint using an MAI elicited significant time-dependent analgesic effects in a mouse model of carrageenan-induced inflammatory pain. Our findings show that changes in TRPV1 expression are closely associated with the anti-nociceptive effects of mechanical acupuncture at ST36, suggesting that TRPV1 signaling may play a role in the observed analgesic response. However, as the data do not clarify whether TRPV1 modulation is a cause or a consequence of pain reduction, we refrain from drawing a definitive mechanistic conclusion. Acupuncture modulates the expression and function of pain-related ion channels (including TRPV1 and NMDA receptors), which can lessen peripheral and central sensitization. Our findings specifically point to an interaction between mechanical acupuncture and the suppression of TRPV1-mediated nociceptive and immune responses. Collectively, these findings provide mechanistic insights into acupoint-mediated analgesia and highlight the therapeutic potential of modulating TRPV1 signaling in the management of inflammatory pain. Collectively, these findings provide mechanistic insights into acupoint-mediated analgesia and highlight the therapeutic potential of modulating TRPV1 signaling in the management of inflammatory pain.

Recent studies have identified TRPV1 as a key mediator of inflammatory pain initiation and persistence. This ion channel is abundantly expressed in C and Aδ primary afferent nociceptors and is sensitized by a variety of endogenous inflammatory mediators, including prostaglandins, bradykinin, and inflammatory cytokines like TNF-α and IL-1β. Sensitization occurs primarily through kinase-dependent phosphorylation and other post-translational modifications that enhance TRPV1 responsiveness under inflammatory conditions [3,8]. This sensitization lowers the activation threshold of TRPV1, resulting in increased neuronal excitability and the release of neuropeptides, such as CGRP and substance P, which further amplify neurogenic inflammation and pain [14]. Consistent with these mechanisms, our results demonstrated that carrageenan injection markedly increased TRPV1 expression in both the spinal cord and plasma. Notably, these elevations were significantly attenuated by MAI treatment or pharmacological inhibition of TRPV1 with capsazepine. The observed suppression of TRPV1 expression following MAI stimulation at ST36 was likely mediated by both peripheral and central mechanisms. The mechanical stimulus appeared to disrupt the acupoint microenvironment and modulate the balance between pro- and anti-inflammatory mediators, thereby directly influencing TRPV1 expression in nociceptors. Concurrently, the reduction in peripheral input was associated with diminished activation of spinal glial cells and attenuation of neuroinflammation, which may have contributed to reduced central TRPV1 upregulation. These findings were consistent with recent reports indicating that transduction induced by acupuncture can downregulate TRPV1 via MAPK/ERK-dependent signaling, suppress cytokine-mediated pathways, and limit neurogenic inflammation [8,12,15]. Importantly, recent studies in both neuropathic and inflammatory pain models have shown that TRPV1 antagonism reduces pain behaviors and attenuates neuroinflammatory responses by suppressing microglial and astrocytic activation in the CNS [8,15]. In our study, both MAI and capsazepine administration suppressed the carrageenan-induced upregulation of the microglial marker Iba1 in the spinal dorsal horn. These findings support the role of TRPV1 as a critical upstream regulator of neuroimmune interactions in pain pathways. Traditional acupuncture and electroacupuncture are well recognized for their analgesic effects, mediated through multiple neurochemical pathways within the nervous system. Han and colleagues demonstrated that opioid receptor antagonists can block acupuncture-induced analgesia, directly implicating endogenous opioids as a key mediator [16,17]. Other studies reveal that adrenergic antagonists attenuate acupuncture’s effects, highlighting its capability to activate descending monoaminergic pathways, particularly norepinephrine and serotonin [11,18]. Furthermore, glutamatergic inhibition—demonstrated by decreased phosphorylation of NMDA receptor subunits—provides another mechanistic link to the observed suppression of central sensitization in pain models following acupuncture [9,19]. Recent research has highlighted the role of ion channels such as TRPV1 and TRPA1 in modulating the effects of mechanical acupoint stimulation, particularly under pathological conditions [20,21]. Our data demonstrated that MAI at ST36 significantly reduced pain-related behaviors and decreased the expression of TRPV1 and pNR2B, a marker of NMDA receptor activation and central sensitization in the spinal cord. In contrast, capsazepine suppressed TRPV1 expression but did not affect pNR2B levels. These results suggest that MAI may modulate both TRPV1-dependent and TRPV1-independent pathways, potentially involving the mechanical disruption of TRPV1/ERK signaling and subsequent attenuation of glutamatergic neurotransmission [15]. Interestingly, the analgesic effects of MAI were highly dependent on both the acupoint and duration of stimulation. Only 60 and 180 s stimulations at ST36 significantly reduced capsaicin-induced paw-licking and carrageenan-induced pain behaviors, whereas shorter stimulation at non-acupoints was ineffective. In rodent models, specific-time stimulation of mechanical acupuncture or electroacupuncture at specific acupoints has been repeatedly demonstrated to induce behavioral analgesia by modulating TRPV1 expression, suppressing glial activation, and inducing adrenergic and opioid responses [9,11]. Similarly, human clinical studies suggest that effective acupuncture sessions often utilize point stimulation within this time range, with diminishing returns for excessive duration [19]. This time dependency is believed to reflect the kinetics of mechanosensitive ion channel activation, neuropeptide release, and local tissue responses at structurally rich acupoints. The translational relevance of these findings is underscored by the growing clinical interest in TRPV1-targeted therapies for pain conditions such as rheumatoid arthritis and orofacial pain, where TRPV1 antagonists have shown efficacy in reducing both pain and inflammation [14]. However, systemic TRPV1 antagonists can produce adverse effects, such as impaired thermoregulation, underscoring the need for localized, non-pharmacological approaches. Our results suggest that MAI offers a promising alternative, providing targeted analgesia through the modulation of peripheral and central TRPV1 activity without systemic side effects. Moreover, the observed suppression of microglial activation by MAI supports the emerging view that neuroimmune modulation is critical for effective pain management. As microglia and astrocytes play distinct roles in acute and chronic pain, future studies should investigate the long-term effects of repeated MAI treatments, as well as the potential synergistic effects with pharmacological TRPV1 antagonists or other neuromodulatory approaches [13,15].

Despite the strengths of this study, several limitations should be acknowledged, including the limited generalizability of the animal model, the exclusive focus on acute pain, and the lack of in-depth mechanistic investigation of relevant pain pathways and cellular contributors. In particular, we acknowledge that, while changes in TRPV1 and glial marker expression are clearly associated with pain behaviors in our model, these findings represent an initial step in elucidating the molecular underpinnings of MAI-mediated analgesia. Additional experiments—including the use of selective pathway inhibitors, conditional knockout models, and live-imaging of neuronal activity—will be required to definitively establish a causal mechanistic link between acupoint stimulation, TRPV1 antagonism, and pain relief. These considerations highlight important directions for future research, such as the incorporation of chronic pain models, comprehensive neuroimmune profiling, and the design of translational clinical studies.

## 4. Materials and Methods

### 4.1. Animals

A total of 90 adult male ICR mice (body weight 20–25 g, obtained from Samtako, Osan, Republic of Korea) were used in this study. All animals were clinically healthy at the time of arrival, free of overt signs of disease, trauma, or infection. Prior to experimental procedures, each mouse underwent routine health screening, including daily observation for activity, appearance, and behavior, according to standardized animal facility protocols. Mice displaying any abnormal findings were excluded from the study. Animals were acclimated to the animal facility for at least one week prior to the experiment and maintained under standardized environmental conditions (24 ± 2 °C, 40–60% humidity, 12/12 h light/dark cycle) with free access to food and water. All experimental procedures were conducted in accordance with the ethical guidelines of the International Association for the Study of Pain and were approved by the Animal Care and Use Committee at the Korea Institutes of Oriental Medicine (reference number 22–052). Efforts were made to minimize animal distress and to reduce the number of animals used, in accordance with the principles of the 3Rs (Replacement, Reduction, and Refinement).

### 4.2. MAI Treatment

Traditional manual acupuncture involves needle insertion into an acupuncture point, sometimes including twisting. Sterile, disposable acupuncture needles (0.18 mm in diameter × 20 mm in length) were used for all procedures. The needles were manufactured by DongBang Acupuncture (DongBang Medical Co., Ltd., Seongnam, Republic of Korea), a GMP-certified company specializing in medical-grade acupuncture equipment. All needles were made of stainless steel and were used only once per animal to ensure sterility and consistency. To mimic the vibrations produced via manual acupuncture stimulation, an MAI was developed by researchers at Daegu Haany University and Korea Institute of Oriental Medicine in South Korea [22]. Unlike conventional acupuncture methods, this device consists of a custom-made control unit and cell vibrator connected to an acupuncture needle. The MAI was set to 1.3 m/s^2^ intensity and 85 Hz for the experiments. All animals were habituated to the experimental procedures, including handling and acupuncture manipulation without needle insertion, for at least 1 week before the study. Stainless steel needles were inserted vertically to a depth of 3 mm into the acupuncture points, and the tails of the animals were lightly restrained by hand. MAI was applied bilaterally at the acupuncture points and tail for 30, 60, and 180 s and maintained for up to 60 s after needle insertion.

The experimental animals in the ST36, PC6, and LI4 treatment groups were treated at ST36 on the lateral side of the tibia, PC6 on the forelimb, and LI4 between the base of the thumb and index (pointer) fingers, respectively. From an anatomical point of view, the ST36 (Joksamli) acupoint is located laterally below the stifle joint adjacent to the anterior tubercle of the tibia [11]. The PC6 (Neiguan) point is located between the palmaris longus and flexor carpi radialis tendons, proximal to the transverse crease of the wrist [23]. The LI4 (Hapgok) acupoint is anatomically positioned on the dorsum of the forepaw, lateral to the midpoint of the second metacarpal bone [24]. The mouse tail (1/3 tail length from the proximal region) was used as a stimulation control site to determine the effect of mechanical stimulation at non-acupuncture points. All acupuncture treatments were performed by a single skilled acupuncturist.

### 4.3. Capsaicin-Induced Spontaneous Pain Test

A capsaicin-induced spontaneous pain model was used to determine the pain-related acupoints and MAI treatment time with optimal anti-nociceptive effects. The capsaicin (Sigma-Aldrich, St. Louis, MO, USA) test was performed as described previously [25]. Each mouse received an intraplantar injection of 20 μL capsaicin solution (1.6 μg/paw) into the right hind paw. Immediately following capsaicin injection, the animals were placed in transparent observation chambers (15 × 15 × 15 cm), and nociceptive behavior was assessed by measuring the duration of paw-licking over a 15 min period. Animals in the vehicle group received an intraplantar injection of physiological saline.

### 4.4. Carrageenan-Induced Inflammatory Pain and Drug Injection

To induce peripheral inflammation, 50 μL of 2% λ-carrageenan (Sigma, St. Louis, MO, USA) in sterile saline was injected into the plantar surface of the right hind paw. Control mice were administered 50 μL of sterile saline intraplantarly into the right hind paw. To minimize distress, intraplantar injections of carrageenan or saline were carried out under light anesthesia using 3% isoflurane in a N_2_O/O_2_ gas mixture.

To investigate the analgesic effects of MAI, 24 animals were randomly allocated into four treatment groups as follows: naïve animals (Normal, n = 6), 2% carrageenan-injected animals (2% CR, n = 6), MAI-treated animals before carrageenan injection (MAI + CR, n = 6), and animals treated with capsazepine intrathecally before carrageenan injection (CZP + CR, n = 6).

For pretreatment, capsazepine (Sigma-Aldrich, St. Louis, MO, USA, 1 μg/mouse) dissolved in physiological saline was administered via intrathecal injection 5 min before carrageenan induction. Intrathecal injections of capsazepine were performed according to the procedure reported in a previous study [23], administered with a 10 μL Hamilton microsyringe fitted with a 30-gauge needle.

Briefly, the animal was gently but firmly held at the level of the iliac crests using the thumb and middle finger, and the fifth lumbar spinous process was identified by palpation with the index finger. Intrathecal injection was performed by inserting the needle into the lumbar 5 to lumbar 6 intervertebral space, with a characteristic tail-flick response confirming successful administration. Capsazepine was administered slowly over a 10 s period. Following the injection, the needle was carefully withdrawn from the spinal cord, and the animals were immediately returned to their observation chambers.

### 4.5. Evaluation of Pain Behavior

Behavioral assessments were carried out in compliance with the ethical standards set by the International Association for the Study of Pain. To evaluate carrageenan-induced peripheral inflammatory pain responses, including mechanical allodynia and thermal hyperalgesia, paw withdrawal was measured using a 0.4 g von Frey filament (North Coast Medical, Morgan Hill, CA, USA) and a plantar analgesia meter (IITC Life Science Inc., Woodland Hills, CA, USA), as previously described [11]. Briefly, baseline measurements of paw withdrawal responses to mechanical and thermal stimuli were obtained before carrageenan administration. Following random assignment to treatment groups, all behavioral evaluations were carried out by blinded investigators at 1, 2, 4, 8, and 24 h post-carrageenan injection. These tests were conducted at the same time of day to reduce errors related to the diurnal rhythm.

The paw withdrawal response frequency to normal innocuous mechanical stimuli was measured using a von Frey filament with a force of 0.4 g. Each mouse was positioned on a wire mesh floor enclosed by a plastic chamber, and mechanical stimulation was applied to the plantar surface of the hind paws using a von Frey filament from below. The von Frey filament was applied 10 times to the hind paw, and the number of withdrawal responses was recorded. Mechanical sensitivity was expressed as the percentage withdrawal response frequency (%), calculated as the number of positive responses out of 10 stimuli. For thermal hyperalgesia assessment, animals were habituated for 30 min in plastic chambers (15 cm diameter × 20 cm length) positioned on a transparent glass floor. Thermal nociceptive thresholds were determined by directing a radiant heat source under the glass platform to the plantar surface of each hind paw. Paw withdrawal latency was recorded with a resolution of 0.1 s using a plantar analgesia meter, with a 20 s cutoff to avoid potential tissue injury. Behavioral testing was carried out by an investigator who was unaware of the group assignments.

### 4.6. Plasma TRPV1 Assays

To assess TRPV1 plasma levels, the animals were anesthetized using isoflurane and decapitated after the carrageenan-induced pain behavior test, and 1 mL of blood was collected in tubes containing EDTA (1.6 mg/mL). Following centrifugation at 3000× *g* for 10 min at 4 °C, plasma was isolated from blood samples for TRPV1 concentration. The TRPV1 levels in the plasma were measured using a commercial ELISA kit (MSB2533461, MyBioSource, San Diego, CA, USA). The values are expressed in ng/mL.

### 4.7. Western Blotting and Image Analysis

Western blotting was performed as previously described [26]. Mice were anesthetized with an intraperitoneal injection of Zoletil 50 (2.5 mg, Virbac Laboratories, Carros, France) and Rompun (0.47mg, Bayer Korea, Seoul, Korea) in saline. The spinal cord was then rapidly extracted by hydraulic extrusion into a chilled saline-filled glass dish and snap-frozen in liquid nitrogen for subsequent analysis. To confirm the anatomical location of the L4–L6 spinal cord segments for Western blot analysis, the attachment sites of individual spinal nerves were identified in anesthetized mice. Each spinal segment was then carefully bisected into left and right halves under a neurosurgical microscope. Subsequently, the spinal cords were further divided into dorsal and ventral regions by making a transverse cut through the central canal and laterally to the midpoint of the white matter. The dorsal horns from both the left and right sides were isolated and used for Western blotting. Lumbar spinal cord segments (L4–L5) were homogenized in RIPA buffer (Cell Signaling, Danvers, MA, USA) supplemented with protease and phosphatase inhibitors, as well as 0.1% sodium dodecyl sulfate (SDS). The homogenates were centrifuged at 12,000× *g* for 20 min at 4 °C to remove insoluble debris. Protein concentrations in the supernatants were determined using the Bradford assay (Bio-Rad Laboratories, Berkeley, CA, USA). Spinal cord lysates were separated by 6% or 10% SDS-polyacrylamide gel electrophoresis and transferred onto nitrocellulose membranes. To block nonspecific binding, membranes were incubated for 30 min at room temperature in T-TBS containing 5% nonfat dry milk (Becton, Franklin & Lakes, NY, USA) and 8% bovine serum albumin (MP Biomedical, Auckland, New Zealand). Membranes were incubated overnight at 4 °C with the following primary antibodies diluted in blocking buffer: anti-β-actin (1:1000, Sigma, St. Louis, MO, USA), anti-TRPV1 (1:500, Santa Cruz Biotechnology, Santa Cruz, CA, USA), anti-phospho-NMDA receptor NR2B (1:1000, Billerica, MA, USA), anti-GFAP (1:1000, Millipore, Billerica, MA, USA), and anti-Iba1 (1:1000, Abcam, Cambridge, UK). After three washes with T-TBS (10 min each), membranes were incubated for 1 h at room temperature with HRP-conjugated secondary antibodies: goat anti-mouse IgG (1:2000, Calbiochem, Darmstadt, Germany) or goat anti-rabbit IgG (1:2000, Calbiochem, Darmstadt, Germany). Immunoreactive bands were visualized using an enhanced chemiluminescence detection kit (Pharmacia-Amersham, Freiburg, Germany). For all Western blot experiments, tissue from each animal was processed and analyzed individually (no pooling was performed). Membranes were independently developed for each sample. After scanning, band intensities were quantified using ImageJ software, version 1.54p (GraphPad Software, Stapleton, NY, USA) and normalized to β-actin for each lane.

### 4.8. Data Analysis

All data are reported as means with corresponding standard error of the mean (SEM). All statistical analyses were carried out using GraphPad Prism version 5.0 (GraphPad Software, Solana Beach, CA, USA). Given the behavioral nature of this preclinical animal study, each experimental group included n = 6 mice. Although larger samples may provide increased power, robust differences in behavioral pain responses and protein expression were detected and confirmed through appropriate statistical testing. Two-way analysis of variance (ANOVA) was applied to assess the effects of treatment and time in behavioral experiments (e.g., mechanical allodynia, thermal hyperalgesia). When significant interactions or main effects were observed, Tukey’s multiple comparison test was used post hoc to determine inter-group differences. For group comparisons in the capsaicin-induced paw-licking test and plasma TRPV1 concentration, we applied one-way ANOVA, followed by Dunnett’s post hoc test for multiple comparisons against the control group. For comparisons between two groups only—such as in Western blot analyses—we used an unpaired two-tailed Student’s t-test. A *p*-value of <0.05 was considered statistically significant.

## 5. Conclusions

In conclusion, our findings demonstrate that mechanical stimulation of the ST36 acupoint using MAI produces time-dependent analgesic effects in a mouse model of carrageenan-induced inflammatory pain. These effects are associated with reduced TRPV1 expression and microglial activation in the spinal dorsal horn, underscoring the central role of TRPV1 in neuroimmune pain modulation. MAI is a promising non-pharmacological strategy for the management of inflammatory pain with potential advantages over systemic TRPV1 antagonists. Further studies employing selective TRPV1 agonists or antagonists, genetic knockout models, or cell type-specific manipulations are needed to clarify whether the observed changes in TRPV1 signaling are causally linked to the analgesic effects of mechanical acupuncture.

## Data Availability

Data is contained within the article.
